# Consensus-Based Guidelines for Best Practices in the Selection and Use of Examination Gloves in Healthcare Settings

**DOI:** 10.3390/nursrep15010009

**Published:** 2025-01-02

**Authors:** Jorge Freitas, Alexandre Lomba, Samuel Sousa, Viviana Gonçalves, Paulo Brois, Esmeralda Nunes, Isabel Veloso, David Peres, Paulo Alves

**Affiliations:** 1 Oncology Medicine Service, Instituto Português de Oncologia do Porto (IPO-Porto), 4200-072 Porto, Portugal; jfreitas@ipoporto.min-saude.pt; 2 Porto Comprehensive Cancer Centre (Porto.CCC), 4200-072 Porto, Portugal; 3 Portuguese Oncology Nurse Association (AEOP), 4200-177 Porto, Portugal; 4 Operating Room Department, Unidade Local Saúde São José, 1150-199 Lisbon, Portugal; alexandre.lomba@ulssjose.min-saude.pt; 5 ICU Unidade Local Saúde Alto-Minho, 4904-858 Viana do Castelo, Portugal; samuel.sousa@ulsam.min-saude.pt; 6 Portuguese Society of Critical Care Nursing (SPEDC), 3030-490 Coimbra, Portugal; 7 Cirurgia Cardiotorácica, Unidade Local de Saúde São João, 1150-199 Lisbon, Portugal; viviana.pinto@ulssaojoao.min-saude.pt; 8 Council Member European Wound Member Association (EWMA), DK-2000 Frederiksberg, Denmark; 9 Operating Room Department, Unidade Local de Saúde do Baixo Alentejo, 7801-849 Beja, Portugal; paulo.brois@ulsba.min-saude.pt; 10 Operating Room Department, Instituto Português de Oncologia do Porto (IPO-Porto), 4200-072 Porto, Portugal; esmeraldanunes@ipoporto.min-saude.pt; 11 Portuguese Association of Operating Room Nurses (AESOP), 1749-008 Lisbon, Portugal; 12 Local Unit of the Infection Prevention and Control and Antimicrobial Resistance Program, Unidade Local de Saúde de Braga, 4710-243 Braga, Portugal; isabel.veloso@hb.min-saude.pt; 13 National Infection Control Association (ANCI), 1749-008 Lisbon, Portugal; davidperes@ulsm.min-saude.pt; 14 Infection and Antibiotic Resistance Control Unit, Unidade Local de Saúde de Matosinhos, 4450-021 Matosinhos, Portugal; 15 Escola Enfermagem (Porto), Faculdade Ciências da Saúde e Enfermagem, Universidade Católica Portuguesa, 4169-005 Porto, Portugal; 16 Centre for Interdisciplinary Research in Health (CIIS)—Wounds Research Lab, 3504-505 Viseu, Portugal; 17 Portuguese Wound Management Association (APTFeridas), 4420-283 Gondomar, Portugal

**Keywords:** healthcare-associated infections, antimicrobial resistance, examination gloves, infection control, sustainability: wound care

## Abstract

Background/Objectives: Healthcare-associated infections (HAIs) and antimicrobial resistance (AMR) present significant challenges in modern healthcare, leading to increased morbidity, mortality, and healthcare costs. Examination gloves play a critical role in infection prevention by serving as a barrier to reduce the risk of cross-contamination between healthcare workers and patients. This manuscript aims to provide consensus-based guidelines for the optimal selection, use, and disposal of examination gloves in healthcare settings, addressing both infection prevention and environmental sustainability. Methods: The guidelines were developed using a multi-stage Delphi process involving healthcare experts from various disciplines. Recommendations were structured to ensure compliance with international regulations and sustainability frameworks aligned with the One Health approach and Sustainable Development Goals (SDGs). Results: Key recommendations emphasize selecting gloves based on clinical needs and compliance with EN 455 standards. Sterile gloves are recommended for surgical and invasive procedures, while non-sterile gloves are suitable for routine care involving contact with blood and other body fluids or contaminated surfaces. Proper practices include performing hand hygiene before and after glove use, avoiding glove reuse, and training healthcare providers on donning and removal techniques to minimize cross-contamination. Disposal protocols should follow local clinical waste management regulations, promoting sustainability through recyclable or biodegradable materials whenever feasible. Conclusions: These consensus-based guidelines aim to enhance infection control, improve the safety of patients and healthcare workers, and minimize environmental impact. By adhering to these evidence-based practices, grounded in European regulations, healthcare settings can establish safe and sustainable glove management systems that serve as a model for global practices.

## 1. Introduction

Appropriately selecting and using examination gloves are critical components of infection prevention and control in healthcare settings. Gloves serve as a primary barrier, protecting healthcare workers and patients from transmitting infectious agents [1,2,3]. However, the misuse or inappropriate selection of gloves can lead to adverse outcomes, including the potential spread of pathogens, skin irritation, or resource wastage. Despite their widespread use, there is significant variability in practice, often influenced by a lack of standardised guidelines or inconsistent adherence to existing recommendations.

Examination gloves are widely used in healthcare settings and, when used alongside proper hand hygiene, are critical too in preventing and combating healthcare-associated infections. They are a fundamental protective measure for both patients and healthcare professionals, helping to mitigate the transmission of harmful pathogens and the emergence of antimicrobial resistance [4]. HAIs increase morbidity and mortality, prolong hospital stays, and increase pressure on the emergency of antimicrobial resistance (AMR) through increased use of antibiotics, thereby increasing health costs [1,4].

According to the Point Prevalence Survey, conducted in 2016–2017 by the European Centre for Disease Prevention and Control (ECDC), HAIs continue to be an important public health problem, with a prevalence of 6.5% and an estimated number of HAI episodes per year in European hospitals is 4.5 million [5]. In view of a risk assessment and the stipulated clinical indications, the use of gloves is a fundamental measure of protection, both for the patient and the health professional, not dispensing hand hygiene before and after use.

The use of protective gloves in healthcare settings is a crucial measure to prevent the transmission of infectious agents and ensure the safety of both patients and healthcare workers [6]. However, the selection and proper use of examination gloves can be a complex issue, requiring careful consideration of various factors [7]. As the implementation of universal precautions has led to a significant increase in the use of gloves for direct patient contact, it is essential to establish consensus-based guidelines for the selection and use of examination gloves to optimize their effectiveness and minimize potential risks [6].

Examination gloves are considered personal protective equipment (PPE) and medical devices (MDs) and are widely used in numerous procedures performed in healthcare. They are the interface between health professionals and patients, so their choice and judicious use are of special importance. However, its widespread availability in the context of care provision has led to a trivialization that has not promoted the critical view that is always essential to the safety of care and its participants.

In light of these facts, the need arose to prepare this document to promote evidence-based good practice (EBP). This document aims to provide detailed consensus-based guidelines on best practices for the selection, use, and disposal of examination gloves in healthcare settings. It is designed to guide healthcare professionals in their daily practice and support decision-making regarding the appropriate use of examination gloves. The development of this document involved collaboration among health professionals from various areas of expertise, which expanded its scope beyond traditional healthcare provision. In this sense, it incorporates the One Health approach and aligns with the Sustainable Development Goals (SDGs), specifically SDGs 9, 12, 13, 14, and 15 [8,9]. By establishing these guidelines, healthcare organizations can help ensure the consistent and effective use of examination gloves, ultimately reducing the risk of healthcare-associated infections and improving the overall safety of patient care.

The guidelines presented in this document are firmly grounded in key European regulatory frameworks, including Regulation (EU) 2017/745 and the EN 455 standard series [10,11,12,13], which define essential quality and safety requirements for medical devices and single-use gloves. These European regulations ensure that gloves meet stringent standards for durability, safety, and efficacy, addressing critical healthcare challenges specific to the region, such as infection control and sustainability. Additionally, the guidelines emphasize the value of European manufacturing practices, which not only uphold high-quality production standards but also contribute to minimizing environmental impact through reduced transportation emissions and recyclable packaging. While the recommendations are shaped by the European healthcare context, they incorporate universal principles of infection prevention and environmental responsibility, making them adaptable and applicable to healthcare systems globally.

## 2. Materials and Methods

The guidelines presented here are informed by the latest evidence, international regulations, and sustainability frameworks, and they were developed through a structured consensus-building process involving healthcare and infection control experts according to ACCORD Guidelines [14].

To establish these consensus-based guidelines, the Delphi method was employed, a systematic approach for gathering and synthesising expert opinions, which is particularly effective in healthcare when defining best practices in an interactive process; this engaged a multidisciplinary panel of 30 experts. These 30 experts were chosen to guarantee a wide representation of specialities and expertise pertinent to the selection and use of examination gloves in healthcare. This panel included specialists in infection prevention and control, clinical and oncology nursing, wound care, perioperative nursing, and medical device standards, among others. Their backgrounds spanned various healthcare sectors, including acute care, critical care, and surgical environments. In addition to their clinical roles, many panel members had significant involvement in national and international organizations, such as the Portuguese Association for Wound Care (APTFeridas), the European Wound Management Association (EWMA), the National Infection Control Association (ANCI), and other professional nursing and public health bodies. This diverse composition ensured that the consensus reflected both frontline clinical perspectives and broader regulatory and sustainability considerations, contributing to the robustness and applicability of the recommendations.

The consensus-building process was conducted over three distinct rounds, each designed to address specific objectives in refining and finalising the guidelines. These rounds enabled thorough evaluation and agreement on key recommendations, ensuring that the final document reflects a robust and evidence-informed consensus.

Round 1: In-Person Workshop

The first round was conducted as a full-day, in-person workshop where all panel members participated. During this session, experts were presented with a comprehensive overview of the current challenges and evidence surrounding glove use in healthcare settings. Discussions were guided by pre-prepared materials, including data on infection control, glove material standards, sustainability concerns, and relevant international regulations such as EN 455. The workshop provided a platform for in-depth deliberations on these topics, allowing panel members to share their insights and experiences. By the end of the day, a preliminary set of key topics and recommendations for glove use were formulated, forming the basis for subsequent rounds of refinement.

Round 2: Structured Feedback

Following the workshop, the second round was conducted remotely. Experts were provided with the draft guidelines derived from the discussions in Round 1 and asked to provide structured feedback using a Likert scale to rate their level of agreement with each recommendation. The scale ranged from 1 (strongly disagree) to 5 (strongly agree). The consensus threshold was set at 75% agreement, consistent with recommendations commonly found in the Delphi methodology literature [15]. Participants were encouraged to provide qualitative feedback alongside their ratings, offering insights or suggesting revisions to further improve the clarity and applicability of the guidelines. The results were aggregated, and areas where consensus was not achieved were identified for further discussion. The time between Round 1 and Round 2 was necessary to allow for the synthesis of the workshop’s findings into a structured draft document for review. This interval ensured that all recommendations were clearly articulated and provided participants with an opportunity to reflect on the discussions and offer more thoughtful feedback during Round 2. To mitigate the risk of participants forgetting key points, detailed workshop notes and summaries were shared with all participants, ensuring continuity and accuracy in the consensus-building process.

Round 3: Final Refinement

In the third and final round, the panel was presented with a revised version of the guidelines, reflecting the feedback from Round 2. This final round focused on refining unresolved points and making minor modifications to reach a full consensus where necessary. Areas that did not initially meet the 75% agreement threshold were re-evaluated, with additional discussion facilitated via digital communication to resolve outstanding issues. By the end of this round, consensus was achieved on all major recommendations, resulting in the final set of guidelines.

This multi-stage Delphi process ensured that the guidelines were both evidence-based and reflective of the collective expertise of the panel while allowing for iterative refinement to achieve broad agreement among participants.

Regarding ethical procedures, no patients or human subjects were involved, and no identifiable data were collected. Our consensus group did not conduct research involving human or animal subjects in the traditional sense. The study was based on a thorough review of existing evidence, regulatory documents, and a consensus methodology (Delphi panel) to establish best practices regarding the use of examination gloves in healthcare settings. Ethical requirements for research involving human participants vary by country. In the country where this consensus was developed, approval by a Research Ethics Committee (REC) is not mandatory for studies involving expert consultation without direct intervention on participants. This approach complies with local ethical regulations governing research activities of this nature. For this reason, Ethics Committee or Institutional Review Board approval was not necessary for this manuscript. To ensure ethical integrity, all participants were fully informed about the purpose and scope of the study and provided their consent to participate. This process aligns with international ethical principles, including respect for autonomy, confidentiality, and transparency. Additionally, all experts who contributed to the consensus panel have their permission to include their names in the publication.

## 3. Glove Selection: Regulation, Criteria, and Considerations

The consensus reached by the expert panel emphasizes the importance of careful selection of examination gloves based on both clinical and environmental factors. Gloves must meet specific criteria for quality, durability, and safety to ensure they provide adequate protection in various healthcare settings.

In Regulation (EU) 2017/745 of the European Parliament and of the Council of 5 April 2017, examination gloves are defined as a medical device (MD), which is “any instrument, apparatus, equipment, software, implant, reagent, material or other article, intended by the manufacturer to be used, alone or together, in human beings, for one or more of the following specific medical purposes: Diagnosis, prevention, monitoring, prediction, prognosis, treatment or mitigation of a disease, …” [16].

EN 455 is the European standard that evaluates the quality and strength of gloves used in the medical industry and under which, “Medical Gloves are intended to be a barrier to agents responsible for transmitting infections to protect patient and user against cross-contamination” [13].

**Scheme 1:**
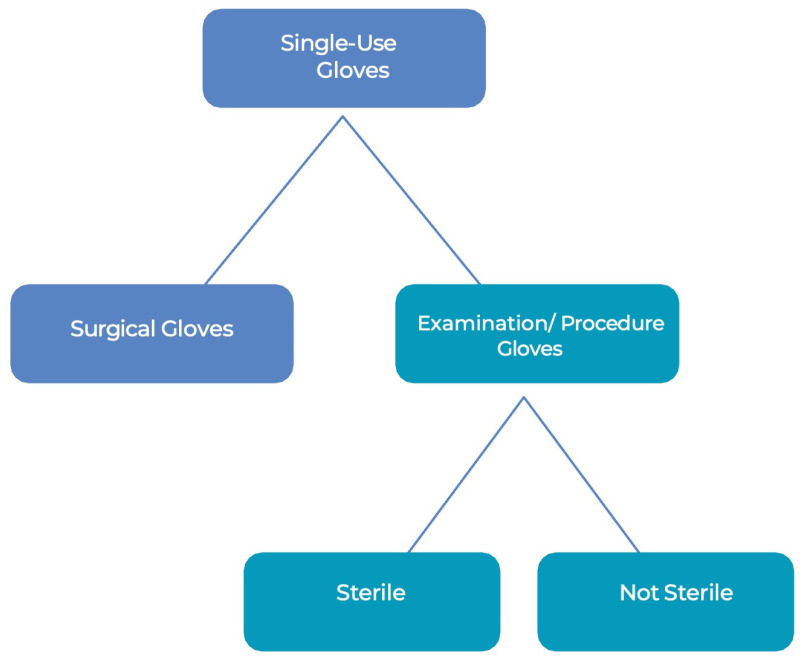
Types of gloves used in the provision of healthcare.

In order to help ensure effectiveness, it is essential that the gloves fit properly in the hand, are free of punctures, and have adequate physical characteristics of strength so that they do not fail during use [13]. This standard defines the following types of gloves (Scheme 1):

The EN 455 standard consists of four parts and defines the tests that examination gloves need to perform by sampling in order to ensure that they comply with the barrier function against microorganisms and that they do not break during their use.

***Part 1: Testing the gloves for the presence of holes***

A leak test is carried out by sampling in which the gloves are filled with one liter of water. This test allows the determination of the Acceptable Quality Limit (AQL) value.

In healthcare, the AQL rating of 1.5 is the upper limit for the batch to be validated under EN 455-1 [10]. To determine the AQL value, it is necessary to submit a sample of gloves to the test described above.

As an example, in a batch of 4,000,000 gloves, the sample to be tested is 500, and 14 gloves may have holes, in which case the entire batch is validated with AQL 1.5.

***Part 2: Physical Property Testing***

The test is carried out to evaluate the force required to break the glove, whose requirement criteria differ according to the raw material and type of glove (Table 1).

***Part 3: Tests for Biological Evaluation The test***

The manufacture of gloves uses various chemicals and microplastics that can cause skin irritations, so tests are carried out to assess the amount of chemical residues present in the gloves.

***Part 4: Determination of shelf life***

A durability test is performed to ensure that the glove does not degrade during transport, storage, and the period while waiting for use. The maximum period of validity allowed is 5 years.

These standards ensure that gloves maintain their protective properties throughout their lifespan, from manufacturing to actual use. The following table (Table 2) outlines the key European Norms (EN) that define the necessary requirements and tests for medical gloves, ensuring their quality, durability, and safety:

## 4. Standards and Recommendations for the Use of Examination Gloves

According to the WHO [14], the use of examination gloves is recommended to reduce the risk of contamination of the hands of health professionals with blood and other body fluids as well as to reduce the risk of dissemination of pathogens to the environment, transmission from the health professional to the patient (and vice versa), and among patients. Examination gloves are single-use and should never be washed, decontaminated, or reused [17].

It is essential to recognise that gloves do not offer comprehensive protection, and healthcare professionals should be aware of this.

Contamination of healthcare workers’ hands can occur due to the presence of defects in the gloves, such as punctures, which facilitate the transfer of pathogens between the hands and the external environment [18].

The risk of contamination of the glove and its packaging is a cause of concern for the scientific community. In a study in an orthopaedic ward about contamination of unused gloves [15], to assess the contamination of examination gloves with pathogens prior to use, it was found that healthcare workers contaminated glove boxes not only with commensal skin microorganisms but also with pathogenic microorganisms, and it was unclear whether the levels of pathogens pose a direct threat to public health. However, previous publications have indicated that the infectious burden for some of these microorganisms is equal to or lower than the level of glove contamination observed in the study, noting that unused and non-sterile gloves are potential carriers of pathogen transmission in hospitals. These results highlight the importance of adhering to hand hygiene guidelines, good practices for removing gloves from the box, and the design of the box as priority targets to reduce contamination of unused examination gloves [19].

The technique for putting on non-sterile examination gloves minimizes the spread of microorganisms responsible for infections, so they should be put on by removing the glove from the packaging, touching only the glove-cuff, and then putting it on without manipulating the glove further.

### 4.1. When to Use Exam Gloves

The proper use of examination gloves in healthcare is a fundamental pillar of good infection control practices, and their use is critical in environments where there is contact with blood and other body fluids, mucous membranes, damaged skin, or potentially contaminated surfaces and equipment [4].

The implementation of good practices involves more than the simple act of putting on and taking off gloves, as it requires hand hygiene, proper handling of this DM/PPE, and identification of situations that require additional protection, so healthcare professionals must have the necessary knowledge and skills to make an informed choice about the selection and use of gloves, to ensure your safety and that of the patients in your care.

Additionally, awareness of the risks associated with reusing or using gloves inappropriately is essential. The practice of reusing gloves or not following proper procedures in removing them can negate the protective benefits, increasing the risk of contamination and cross-transmission [20].

The training of caregivers, family members, and patients on the correct use and removal of personal protective equipment becomes imperative, especially considering that even highly trained health professionals can make mistakes in this process [21]. Thus, providing education about these practices is crucial to ensure safety within the healthcare environment and significantly minimize the risk of adverse events resulting from contamination [22].

### 4.2. Sterile Gloves vs. Non-Sterile Gloves

In healthcare, the proper selection between sterile and non-sterile gloves is a decision that directly impacts infection prevention and the safety of patients and healthcare professionals. This choice should be guided by an understanding of the specific characteristics and needs of each procedure, the risk of infection, and the specific circumstances of the user. The implementation of clear and evidence-based guidelines is essential to guide health professionals in their decision-making and contributes to the optimization of resources, ensuring maximum safety and effectiveness of healthcare.

In the context of chronic wounds, the presence of bacterial colonization is a common reality, where their bed adjusts to the microbial environment. This adaptation may offer some protection against the invasion of pathogens [23]. The persistent nature of these wounds and their prolonged exposure to microorganisms makes the use of non-sterile gloves a practice considered by many to be safe, especially in procedures where absolute sterility is not possible or necessary. Several studies [24,25] argue in favour of the use of non-sterile gloves in chronic wound care, noting that under certain conditions, no significant increase in the incidence of infection was observed. This practice has been shown to be safe in many cases, and the cost reduction associated with the choice of non-sterile gloves, when appropriate, can allow the reallocation of financial resources, thus improving the standard of care provided [26,27].

### 4.3. Selection and Safe Use of the Examination Glove

The selection and use of examination gloves in healthcare depends on several factors. To support the decision of health professionals, Table 3 and Table 4 present the parameters and fundamental factors for the choice of examination gloves (Table 3).

There are also other parameters to consider regarding their quality and protection (Table 4).

### 4.4. Hand Hygiene

Performing hand hygiene before and after the use of gloves is essential in the prevention of infection, with strong scientific evidence. It is a simple measure, represents the first line of defence against the spread of pathogenic microorganisms, and is widely recognised by the scientific community as the key intervention in the fight against HAIs.

Gloves are not foolproof and do not eliminate the need for hand hygiene, and as such, complement but do not replace this essential practice.

Hand hygiene is a widely accepted key strategy in the prevention and control of HAIs, as contaminated hands of healthcare workers are the vehicle most often implicated in the cross-transmission of pathogens [22]. Studies consistently demonstrate that strict adoption of proper hand hygiene practices can significantly reduce the incidence of HAIs [4,29,30,31].

The underlying principle of this procedure is the physical removal and inactivation of microorganisms present on the skin, preventing their transmission to the patient or surfaces in the care environment. The impact of hand hygiene extends beyond the individual protection of the healthcare professional, positively influencing patient safety and the overall quality of care. Implementing hand hygiene practices before and after wearing gloves is a demonstration of commitment to the highest standards of care, reflecting a culture of safety that prioritizes patient well-being.

Investing in hand hygiene education and training, and in the selection and proper use of gloves, promotes safety and quality of care and reduces costs associated with HAI treatment, being a valuable effort for the health system as a whole.

### 4.5. Correct Technique for Putting on and Removing Gloves

The correct technique of putting on and removing gloves is essential to ensure the safety of healthcare workers and effectiveness in preventing the transmission of infections.

Gloves should be removed from the packaging, ensuring that contact is made only through the cuff of the glove and then putting it on, without further handling the glove, so as to completely cover the hand without touching the external surface, thus avoiding excessive manipulation to prevent the transfer of microorganisms from the hands to the glove [13].

After the health procedure has been performed, the glove removal technique assumes critical importance to avoid contamination of the hands. Initially, the user should pinch the outside of one of the gloves at the level of the wrist, ensuring the skin remains untouched. This movement should be performed carefully, pulling the glove out and inverting it during the process. This action prevents any contamination present on the outside of the glove from coming into contact with the skin or dispersing in the environment. When proceeding to remove the second glove, the following technique should be used:

The user should insert their fingers under the edge of the glove into the opposite hand, using the already ungloved hand, and pull it out, ensuring that it also reverses dur-ing removal. The technique requires special attention to not allow direct contact of clean hands with the glove's contaminated surface. This methodology ensures that contamina-tion is not transferred to hands or the environment while maintaining effective control over the potential spread of microorganisms.

The next step is the proper disposal of the removed gloves and hand hygiene. Adherence to this practice reinforces the importance of maintaining a high standard of hygiene in healthcare.

## 5. Recommendations

This consensus document provides evidence-based, practical recommendations for healthcare professionals regarding the selection, use, and disposal of examination gloves. The guidelines aim to improve infection prevention, enhance patient and healthcare worker safety, and support environmental sustainability.

Key Recommendations on Glove Selection and Usage:

- Glove Selection
  - Compliance Standards: Choose gloves that meet EN 455 standards for quality and barrier properties.
  - Acceptable Quality Limit (AQL): Select gloves with an AQL of <1.5 to reduce risks of punctures and contamination.
  - Types of Gloves:
    - Sterile Gloves: For surgical and invasive procedures to maintain aseptic conditions.
    - Non-Sterile Gloves: For routine care with blood, bodily fluids, or contaminated surfaces.
- Usage Best Practices
  - Hand Hygiene: Perform hand hygiene before donning and after removing gloves.
  - Proper Usage: Avoid reusing gloves or wearing them improperly.
  - Education: Ensure healthcare professionals are trained in correct glove donning and removal to prevent cross-contamination.
- Disposal and Sustainability
  - Disposal: Follow local clinical waste management protocols for used gloves.
  - Sustainability: Encourage the use of recyclable or biodegradable gloves, aligning with One Health and Sustainable Development Goals (SDGs) 12 and 13.
- Environmental and Safety Considerations
  - Manufacturing: Prefer gloves made in controlled environments to reduce contamination.
  - Packaging: Use recyclable and watertight packaging to maintain glove integrity and minimize waste.

## 6. Conclusions

The prevention and control of HAIs have become a growing concern among health professionals. Since contaminated hands are the vehicle most frequently implicated in the cross-transmission of pathogens, hand hygiene is a fundamental strategy in the prevention and control of HAIs. However, in several clinical situations, the use of examination gloves is mandatory to protect the health professional and the patient, not dispensing with hand hygiene before and after use.

The use of examination gloves is not without risks and is often related to their quality, ultimately impacting healthcare professionals and the patients they care for. Wearing quality gloves during care is one of the ways to prevent the transmission of HAIs and, consequently, to contribute to the reduction in AMR. On the other hand, examination gloves that remain intact throughout the procedure are synonymous with less waste, thus contributing to the reduction in environmental impact.

These consensus-based guidelines aim to provide detailed recommendations on the best practices for the selection, use, and disposal of examination gloves in healthcare settings. Best practices emphasize selecting gloves based on clinical needs, such as the risk of exposure to blood, body fluids, or infectious materials, and ensuring they meet quality standards like EN 455. Gloves should be used in situations where they provide a necessary barrier, including contact with mucous membranes, non-intact skin, or contaminated surfaces. Proper hand hygiene before and after glove use remains critical to infection prevention.

Disposal practices should follow established protocols to minimize environmental impact. Used gloves must be discarded in designated clinical waste containers, adhering to local regulations for medical waste management. The promotion of sustainable practices, including the use of recyclable or biodegradable glove materials where feasible, aligns with the One Health approach and supports global Sustainable Development Goals (SDGs) 9, 12, 13, 14, and 15. By adhering to these guidelines, healthcare professionals can make informed decisions that enhance patient safety, protect healthcare workers, and minimize environmental impact. Although there is a European focus, the principles of these guidelines can also inform best practices globally.

## Figures and Tables

**Table 1:** Resistance criterion that gloves must comply with, according to EN 455.

Glove Type	Force Needed to Break Through
Surgical gloves	9.0 Newtons
Rubber gloves (nitrile, latex)	6.0 Newtons
Thermoplastic gloves (vinyl, polypropylene)	3.6 Newtons

**Table 2:** Summary of parameters tested according to EN 455.

Norm	Standard Designation	Test Parameters and Their Definitions
EN 455-1 [10]	Single-use medical gloves—Part 1: Requirements and tests for no holes	According to the standard, the examination glove cannot have AQL > 1.5 to be considered accepted. The higher the AQL value, the lower the quality and safety of the glove.
EN 455-2 [11]	Single-use medical gloves—Part 2: Requirements and tests for physical properties	It specifies requirements and provides test methods for physical properties to ensure that gloves present and maintain an adequate level of protection against cross-contamination for the patient and wearer. The standard stipulates that the examination glove must withstand a strength equal to or greater than 6 N (nitrile or latex) or 3.6 N (vinyl or polyethylene). The higher the value of newtons that the glove resists, the more resistant and greater the protection that the glove offers. Tests covered: dimensions (length and width); breaking force.
EN 455-3 [12]	Single-use medical gloves—Part 3: Requirements and tests for biological evaluation	It specifies requirements and provides methods for the assessment of biological safety, such as chemicals, endotoxins, leachable proteins, powder.
EN 455-4 [13].	Single-use medical gloves— Part 4: Requirements and Tests for Determining Shelf Life	Specifies test requirements for determining shelf life.

**Table 3:** Fundamental parameters in the selection of gloves.

Fundamental Parameters	
Parameters	Justification
AQL value < 1.5, according to EN 455-1 [10].	The lower the AQL value, the greater the barrier effect of the glove.
Tensile strength > 6 Newton, according to EN 455-2 [11].	The higher the tensile strength value, the greater the strength of the glove.
Glove cuffs oriented towards opening, according to the WHO Glove Use Information Leaflet.	Compliance with the WHO recommendation.
Sanitizable packaging, since, being a frequently touched surface, it must be cleaned, according to the recommendations of the CDC and Healthcare Infection Control Practices Advisory Committee (HICPAC).	Sanitization with appropriate cleaning agents is essential for infection prevention and control in a healthcare environment.
Glove that can be removed one by one, according to the WHO’s Glove Use Information Leaflet.	Minimizing the manipulation of the gloves allows you to minimize the possibility of cross-contamination.

**Table 4:** Parameters that promote glove quality.

Parameters That Promote Quality	
Parameters	Justification
Recyclable and/or recycled packaging	Decreases the impact of the ecological footprint caused by medical waste.
European production	Decreases the ecological footprint due to transportation. Strong component in social responsibility.
Packaging is watertight until opening in a clinical setting	The watertight packaging protects the gloves from external contamination between the time of production and use.
Gloves Inspection	Decreases the number of defective gloves and decreases the amount of waste produced—One Health approach. It saves time for healthcare professionals.
Particulate-free packaging	ISO-14644-5-2004 standard [28]: control the number of particles present in the environment in clean rooms.
Production in a controlled environment	Minimizes contamination of gloves in the production phase.

## Data Availability

The data presented in this study are available on request from the corresponding author.

## Public Involvement Statement

No public involvement in any aspect of this research.

## Guidelines and Standards Statement

This manuscript was drafted against the ACCORD Guidelines.
